# Assessment of therapeutic response in patients with metastatic skeletal disease: suggested modifications for the MDA response classification criteria

**DOI:** 10.1038/sj.bjc.6605825

**Published:** 2010-07-27

**Authors:** V Vassiliou, D Andreopoulos

**Affiliations:** 1 Department of Radiation Oncology, Bank of Cyprus Oncology Centre, 32 Acropoleos Avenue, 2006, Strovolos, Nicosia, Cyprus; 2 Department of Radiology, Bank of Cyprus Oncology Centre, Nicosia, Cyprus

**Sir,**

We have read with great interest the article published in your journal titled ‘tumor response interpretation with new tumor response criteria *vs* the World Health Organization criteria in patients with bone-only metastatic breast cancer’ (Hamaoka *et al*, 2010). In this article the therapeutic response of breast cancer patients with bone metastases was assessed by applying both the new MDA response criteria (Hamaoka *et al*, 2004) that are based on computed tomography (CT), magnetic resonance imaging (MRI), plain radiography (XR) and skeletal scintigraphy (SS), and the old World Health Organization (WHO) response criteria that are based on XR and SS (WHO, 1979). The authors concluded that the MDA classification system is superior to that of WHO in differentiating between treatment responders and non-responders. We are in total agreement with the authors that large prospective studies should be carried out to establish the MDA criteria for evaluating the therapeutic outcome of patients with bone metastases. This need becomes more urgent if we consider that the well-known Response Evaluation Criteria for Solid Tumors (Therasse *et al*, 2000) do not include bone response evaluations. Moreover, classifications such as the ones of the WHO and the Union International Against Cancer (Hayward *et al*, 1977) were described 30 years ago and do not include modern imaging techniques such as the MRI, CT or positron emission tomography.

We would like to go a step further and comment on the MDA classification response criteria as it is our opinion that they may be improved by becoming more objective and accurate. Starting with the application of CT for assessing bone metastases, it would be very useful if the bone density in regions of metastases is measured in Hounsfield units (HU) after delineation of affected bone areas (region of interest technique, ROI). By comparing bone density measurements before, during and after treatment (Vassiliou *et al*, 2007a, 2007b; Chow *et al*, 2004), researchers would be able to monitor changes of bone density objectively and follow the therapeutic response more accurately.

For osteolytic lesions an increase in bone density up to at least the level of bone density of neighbouring healthy bones would mean a complete response, whereas an increase in absolute numbers would indicate a partial response. In the case that bone density remains unaltered, stable disease should be considered. Finally, in the event that bone density decreases, disease progression should be indicated. In the case of osteoblastic lesions a decrease in bone density up to at least the level of bone density of neighbouring healthy bones would mean a complete response, whereas a reduction of bone density would mean a partial response. As in the case of lytic bone lesions, if bone density remains unchanged, stable disease should be considered, whereas when there is an increase in bone density, disease progression should be indicated. The MDA response criteria are not so objective as they are rather descriptive and do not use specific quantitative measurements. For example, a complete response is considered to be a ‘normalisation of osteoblastic lesion’ or a ‘complete fill-in or sclerosis of a lytic lesion on CT’. A partial response is considered to be a ‘partial fill-in or sclerosis of a lytic lesion on CT’ or a ‘regression of a measurable blastic lesion on CT’, whereas in cases that no changes are observed on CT, stable disease is indicated.

Comments on the use of MRI for assessing the therapeutic response of bone metastases in association with the MDA criteria also aim to improve objectivity. This could be improved by measuring and monitoring absolute or percentage changes of signal intensities in delineated bone metastases (ROI) after applying a specific MRI sequence with or without contrast enhancement. As in the case of CT, this could be carried out before treatment as well as during and after completion of therapy for comparative purposes. A number of studies that used this methodology have been published (Vassiliou *et al*, 2007a; Brown *et al*, 1998; Ciray *et al*, 2001; Montemuro *et al*, 2004) and for the sake of discussion we present only one. In the study by Vassiliou *et al*, patients with bone metastases were evaluated with MRI before and 3 months after the onset of radiotherapy combined with monthly bisphosphonate infusions. At 3 months signal intensities of T1TSE images (with and without gadolinium (Gd) enhancement) were significantly lower than corresponding baseline values (*P*<0.001), indicating a response to therapy. More specifically, Gd enhancement was associated with a 57% increase at baseline as compared to only 15% at 3 months.

Both the measurement of bone density (CT) and signal intensity (MRI) of bone metastases after ROI delineation are effaceable and have been successfully used to evaluate the therapeutic response of patients with metastatic bone lesions. Their probable incorporation in the MDA criteria would improve accuracy and objectivity.
